# Recruiting for Epigenetic Research: Facilitating the Informed Consent Process

**DOI:** 10.1155/2013/935740

**Published:** 2013-06-06

**Authors:** Nancy Jallo, Debra E. Lyon, Patricia Anne Kinser, Debra Lynch Kelly, Victoria Menzies, Colleen Jackson-Cook

**Affiliations:** ^1^School of Nursing, Virginia Commonwealth University, P.O. Box 980567, Richmond, VA 23298, USA; ^2^Department of Pathology, Virginia Commonwealth University, Richmond, VA 23298, USA

## Abstract

Because the effects of epigenetic (gene-environment interaction) changes have been associated with numerous adverse health states, the study of epigenetic measures provides exciting research opportunities for biobehavioral scientists. However, recruitment for studies focusing on any aspect of genetics poses challenges. Multiple factors, including lack of knowledge regarding a research study, have been identified as barriers to recruitment. Strengthening the informed consent process through extended discussion has been found to be effective in recruiting for research studies in general, yet there is a paucity of information that focused on such a recruitment strategy for epigenetic studies. In this paper, we share our experiences with strategies to strengthen the informed consent process as well as provide samples of materials developed to heighten potential participants' understanding of epigenetics, in 4 epigenetic research studies with women from diverse backgrounds experiencing a range of health issues. The combined enrollment success rate for epigenetic studies using the process was 89% with participants representing a diverse population. We posit that carefully developed recruitment scripts provided a foundation for improving potential participants' understanding of the research project. Easy to understand illustrations of the epigenetic process provided a basis for active engagement and encouraged individual questions.

## 1. Introduction

Recruitment, the process of identifying and enrolling eligible participants in a research study, is a fundamental component of all clinical research endeavors [1, 2]. While there are published reports identifying strategies to identify potential research participants for research studies in general [3–10], the published literature focusing on recruiting specifically for genetic research in the USA is limited [11–16], and there is a paucity of studies specific to recruitment and enrollment strategies in epigenetic research. 

This gap in the literature poses a significant problem given the importance of epigenetic research. In the past few years, our understanding of the causal factors leading to complex diseases has expanded to include not only the recognition of causal factors due to an individual's DNA sequence (including polymorphisms), but also the identification of epigenetic changes that result from environmental/social experiences (referred to as gene—environment interactions). This paradigm shift has resulted from a better understanding of epigenetic factors that are involved in human health and illness [17]. Epigenetics is the study of the changes in gene expression, the mitotic inheritance of patterns of gene expression, and nuclear inheritance which is not based on changes in DNA sequence [18]. These additions to the understanding of the genomic revolution have demonstrated the importance of studying the influence of the environment on (epi) genetic mechanisms [19]. Epigenetic changes can switch genes on or off and determine which proteins are transcribed [17]. Environmental factors, in the context of both the external and internallymodulated environment, such as behavior patterns, nutrition, stress, and toxins/pollution, can influence gene expression [19, 20]. The deleterious effects of these epigenetic changes have been associated with diverse conditions including but not limited to, accelerated aging, heart disease, diabetes, obesity, cancer, and mental disorders [21]. Therefore, for biobehavioral scientists, the study of epigenetic measures provides exciting opportunities to identify meaningful relationships among potentially modifiable environmental and life-style variables, with the potential for the future development of targeted interventions to improve health outcomes. 

Epigenetic measures, such as DNA methylation and histone modification, have only recently been incorporated into human clinical research. While there are many potentially important reasons for examining these measures in clinical studies, the process of explaining these concepts and measures to potential study participants is compounded not only by the complexity of the concepts but also the shift in the understanding of genetics from a “gene-centric” view of the former genetic paradigm [22] to the “phenotypic plasticity” of a given genotype to produce different phenotypes in response to different environments [23].

Although there are still many unknowns in the integration of epigenetic measures into clinical research, future knowledge development depends on beginning to include these measures in research studies. In human research, recruiting potentially eligible research participants is one of the key challenges. However, recruiting and enrolling eligible participants for research focusing on any aspect of genetics pose particular challenges. Multiple factors have been identified that can adversely affect the rate of participant accrual into clinical research. For example, mistrust, health status, language, literacy, and lack of knowledge regarding a research study have been identified as barriers to recruitment [5]. These barriers may be even more magnified in low income, minority, or otherwise vulnerable populations resulting in further difficulties to include diverse participants [24]. Consequently, investigators conducting research of a genetic nature may experience even more challenges than other fields regarding the underrepresentation of vulnerable and/or minority populations [25, 26].

One avenue for decreasing potential barriers and improving recruitment is to strengthen the informed consent process. This could be accomplished by considering the cultural and contextual issues that surround the potential participant's reactions to informed consent, the comprehension of information delivered during the process, and the ability of researchers to address these issues [27]. There is evidence to suggest that such a strategy is potentially effective when recruiting for research studies in general [28], yet there is a paucity of published literature that focused on such a recruitment strategy for epigenetic studies. Obtaining informed consent is an essential process by which potential study participants are presented with the opportunity to make informed decisions about their participation in research [27]. An informed consent document should always include an explanation of the study, a description of risks, benefits, and confidentiality, methods to contact the investigators in the event of adverse events or questions, a statement that participation in the study is voluntary, and procedures for withdrawing from the study [29, 30]. However, this document alone does not typically allow for interested individuals to share their thoughts, feelings, or concerns about participating in the study. In addition, the Declaration of Helsinki suggests that the well-being of study participants should be the priority of the researcher, thus it is important to ensure that potential participants are comfortable talking with researchers about any concerns [31]. It is prudent to view informed consent as having two components: (1) the written information shared and signed by all participants and (2) the process by which the research team provides the information and answers questions [32]. 

 Often the focus of informed consent may be on the actual signing of the consent document rather than on the interaction between the researcher and participant to ensure that communication and understanding have been achieved [27]. This focus seems ill-advised, as standard consent forms with their technical language, multiple sections, and length can impede a participant's understanding [33, 34] which, in turn, may undermine the recruitment and retention of participants [35]. While limited, there is evidence to suggest that additional information provided to potential participants outside the formal consent document may increase their knowledge and have a positive effect on participant recruitments for several types of studies, including clinical trials involving patients with advanced cancer [36]: African American outpatients [37], pregnant women [38], and women being screened or treated for breast cancer [39]. These results provide support for the premise that strengthening the informed consent process, through an increased understanding of information, may be a potential avenue for improving unbiased recruitment [27]. 

In this paper, we share our experiences with strategies to strengthen the informed consent process as well as provide samples of materials developed to heighten potential participants' understanding of epigenetics, in 4 epigenetic research studies with women from diverse backgrounds experiencing a range of health issues. As nurse scientists at the Virginia Commonwealth University School of Nursing, we are investigating relationships between epigenetic changes and psychobiologic symptom measures in women of varying ages, ethnicity/race as well as health status, and we have recognized the potential benefit of strengthening the informed consent process.

*Aim.* This paper is a process evaluation of the extended discussion strategy the authors developed and implemented to enhance recruitment enrollment in 4 separate research studies. Therefore, the aim of this paper is to (1) describe the development and implementation of an expanded informed consent process; (2) provide samples of the recruitment materials developed to heighten potential participants' understanding of epigenetics; and (3) report the overall enrollment rate in the 4 studies using this process.

## 2. Materials and Method

### 2.1. Design

After reviewing the literature to identify which strategies may be most effective in improving potential participant's understanding of the research study, we concluded that a successful consent process is likely to require one-to-one interaction with someone knowledgeable about the study [28, 40]. Extended discussion between the research team and potential study participants has resulted in statistically significant increases in participants' understanding [41–43]. For example, patients having cancer who received a standard informed consent procedure supplemented with a telephone-based informational contact session by a nurse were found to be significantly more informed about a research study compared to those receiving standard informed consent procedures from the treating physician [41]. Similarly, in a study among poor, urban potential research participants, comprehension of the consent form increased following extended discussion with a counselor before meeting with the study investigator [43]. Also, cardiac patients demonstrated an increase in understanding with repeated education about the research protocol [42]. In addition, discussions with the research team to review the research and informed consent document were found to be preferred in 97% of low-income and minority women compared to reading it themselves [44]. Taken in total, these studies provide evidence to suggest that extended person-to-person interactions and discussions enhance participant understanding. However, no similar strategy has been used nor evaluated in epigenetic research, a topic of increased interest to many researchers. To address this gap, the authors developed and implemented research materials designed to heighten eligible participants' understanding of epigenetics to strengthen the informed consent process. 

Several other factors were considered in the development of the recruitment materials. We acknowledged that the language of epigenetics might be difficult for the layperson to understand. Words used in epigenetic research, such as epigenetics, telomeres, and DNA methylation, are most likely unfamiliar to potential study participants and may be confused with genetic research. To meet this challenge, we developed a basic recruitment *script* that strived to use “plain language” or “plain English” that is straightforward and easy to understand [45]. It would come as no surprise that information delivered in plain language appeals to patients [46] and is related to increased satisfaction and decreased anxiety with the informed consent process [18]. In addition, we employed metaphors to clarify potentially unfamiliar concepts since every day examples can be valuable tools of communication when attempting to clarify complex concepts [47]. For example, metaphors such as “our bodies are made up of cells, much like a beach is made up of small grains of sand” or “genes can be turned on and off just like lights in your house can be turned on and off” were added to increase understanding. The initial script was reviewed by experts in health policy and the national genetic service sector, as well as African American, Hispanic, and Caucasian women of ages similar to potential study participants. The script was then revised based on their comments. 

The basic script served as the framework for the extended discussion but was tailored to meet the experiences and concerns of potential participants from diverse backgrounds [48]. For example, with potential participants who were pregnant, the researcher added to the basic script assurances that the study was not looking for, or testing for, any genetic problem in the baby. An example of the basic recruitment script is included (Appendix A). 

Epigenetic recruitment scripts were supplemented with a list of Frequently Asked Questions (FAQs). The contents of this list were based on comments from the reviewers of the script as well as feedback from researchers experienced in epigenetic research. The proposed answers to the FAQs were designed to be straightforward and also included metaphors. For example, when asked “can you find anything bad,” the proposed answer was intended to reassure the potential participant. The answer that stated “We are not looking at inherited changes that cause people to have genetic or inherited diseases. We are looking at things that might turn genes on and off in our body's cells. To use our house analogy, we are not looking at how the wiring in the house is designed, but rather changes in how the appliances plugged into the wiring are used” may help the potential participant to understand the goal of the research. The FAQs were viewed as tools to assist the research team in addressing questions or issues that may arise. Samples of Frequently Asked Questions are provided (Appendix B). 

### 2.2. Research Studies and Participants 

The cohort for this report of our epigenetic informed consent process was created by combining the recruitment and data collection efforts of 4 individually funded research studies. While separate, each study is related through the epigenetic aim.  Table 1 summarizes the aims, target population, and recruitment goals of the four studies. The author researchers incorporated the recruitment materials into their individual study protocol. Participants for the 4 studies were recruited from health care providers' offices and the community in an urban metropolitan as well as rural area in the Southeast area of the United States. Participants in previous research studies who had expressed interest in future studies were also contacted. Recruitment methods included use of brochures and flyers placed in physician offices, ambulatory care clinics, and churches; newspaper ads; referrals from clinical and community partners; and face-to-face personal recruitment. Depending on the research design, person-to-person extended discussions occurred either face to face or over the phone and typically lasted for 15–20 minutes. 

### 2.3. Data Analysis

Descriptive statistics were used to analyze demographic data and calculate the enrollment rate or recruitment yield. The recruitment rate represents the number of participants enrolled divided by the number of participants eligible for the study. 

### 2.4. Ethics

Ethical approval for all studies was obtained from the Virginia Commonwealth University Institutional Review Board. 

## 3. Results

### 3.1. Recruitment Demographics and Yield

The cohort for this paper, which is a reporting of our process evaluation, is a convenience sample of 102 women enrolled in one of the four epigenetic studies. The participants ranged in age from 18–71 years. The mean age of participants was 46. The majority of the sample was Caucasian (*n* = 61/102) which is 60% of the total sample. African American participants represented 38% of this sample (39/102). The total enrollment for African American women from the urban center for this study is greater than 50% (16/31). The combined sample also includes 1% Asian participants and 1% of the participants are Hispanic. The average success rate for enrollment for these studies combined to date (*n* = 102) was 89%. The findings are provided in Table 2. 

### 3.2. Participant Comments

Of the eligible participants who decided not to enroll, the majority cited the following reasons for not enrolling: (1) feeling overwhelmed by the situation of having cancer and the treatment schedules; (2) concern over being anemic and having additional blood being drawn; (3) not wanting blood drawn at time of study since “just stuck yesterday at the doctor's office.” No potential eligible participant stated concern over the concept of epigenetics or aims of the study as reasons for refusal.

## 4. Discussion

The aim of this report of our epigenetic recruitment process was to describe the development and implementation of recruitment materials developed to expand the informed consent process to heighten potential participants' understanding of epigenetics. We report the overall enrollment rate in the studies using these tools as a potential proxy measure for their efficacy. Because there is a paucity of epigenetic recruitment literature, we frame our discussion within the context of genetic research recruitment. 

The overall rate of enrollment was 89% using our enhanced informed consent process, higher than reported rates from other genetic studies conducted in the USA which ranged from 15.9% to 84.9% ([13, 15, 16, 49–51]. For example, researchers recruiting adults with temporal lobe epilepsy reported that, of the eligible patients, 15.9% enrolled in genetic studies [13]; another study reported that 57% of the mostly Caucasian potential participants consented [49]; 62.3% of eligible African American and Latinos participated in a lung cancer study [15]; the Agricultural Health study obtained informed consent from 79% of those male farmers contacted [50]; and the NHAMES study reported that 84.8% in the 2000 survey of eligible participants representative of the general population consented to have DNA stored in a national repository [51]. The rate of enrollment in this study was slightly lower than that in the Action for Health in Diabetes clinical trial which reported an overall enrollment rate of 89.6% across 15 institutions conducting DNA research among overweight or obese individuals with type 2 diabetes [16]. 

Participation in research is often lower among women in general as well as members of minority racial/ethnic groups, which may pose a challenge when recruiting for epigenetic research [14, 16]. For example, females and non-Hispanic blacks consistently had lower consent rates compared to representative sample of the USA population [51]. Similar findings were reported by Espeland et al. [16] who found nonconsent occurred more frequently among African-American, Hispanic, and female potential participants. We did not encounter this phenomenon. Participants in this cohort study represented a diverse population of Caucasian, African American, Asian, and Hispanic participants. Study participants from minority ethnic/racial groups comprised 40% of our combined cohort, which represents the profile of our community's population (U.S. Census Bureau). The ethnic/racial categories of our participants are presented in Table 2.

 Due to several study limitations, the high enrollment rate cannot be attributed solely to the efficacy of the expanded discussion and recruitment materials. Because there was no randomization, the high rate of enrollment could be the result of factors other than the expanded informed consent process. The small sample size of the pilot studies limits generalizability. In addition, the willingness of these women to join an epigenetic study may be attributed to the prior experience and the established relationships between the potential study participant and the researcher [9]. Also, because there were 4 separate studies with different research teams, the possibility of recruitment variance cannot be ruled out. While the recruitment materials did provide consistency of information delivered, individual interactions with eligible participants may have influenced enrollment [52].

While our results must be balanced with study limitations, we suggest that the materials we implemented to improve the informed consent process contributed to our success in recruiting diverse samples for our studies. We posit that carefully developed recruitment scripts provided a foundation for improving potential participants' understanding of the research project. Easy to understand illustrations of the epigenetic process provided a basis for active engagement and encouraged individual questions. The frequently asked question section was a way for prospective participants to understand that it was “normal” to have questions and to want more information about a specific research study in which they were eligible for enrollment. Using familiar words and ideas to simplify information and teach potential participants is considered an important component to successful participant recruitment [45].

The basic recruitment script, as well as the FAQs document, established consistency among members of the research team, encouraged engagement in conversation with each participant to ensure understanding, and provided an opportunity to address participants concerns. We suggest that by incorporating the FAQs and the extended discussion into our individual protocols, we may have positively impacted recruitment into our various epigenetically focused research studies; thus this may be a potential strategy for strengthening future informed consent processes. 

## 5. Conclusion

Recruiting for genetic and epigenetic studies will be an ongoing challenge for scientists conducting research in human subjects. We believe that expanding the information and dialogue involved in the informed consent process is a foundational step for further participants to accept of their active engagement in research studies. In epigenetic studies, it is important to view informed consent as a process that depends on interactions between the researcher and a potential participant. Continued success with implementing biobehavioral research depends on the careful development of strategies that create a safe and open environment for dialogue among research scientists and potential research participants. 

## Figures and Tables

**Table 1:** Overview of studies.

Research title	Epigenetic aim	Population	Recruitment goal
Epigenetics and psychoneurologic (PN) symptoms in women with breast cancer (Lyon et al.)	(1) Longitudinally examine levels of inflammation and epigenetic patterns pre-, peri-, and postchemotherapy (2) Longitudinally examine relationships among inflammation, epigenetic changes, and PN symptoms	Women diagnosed with early-stage breast cancer	*N* = 75

Epigenetics and maternal stress (supplement to ongoing intervention trial) (Jallo and Cook)	Longitudinally examine relationships among epigenetic changes, stress, anxiety, depression, and stress biologic markers	Low-income healthy pregnant women	*N* = 10

Stress, immunity and symptoms in Fibromyalgia (Menzies and Cook)	Examine relationships among stress, inflammation, environment, fibromyalgia symptoms, and gene expression	Women with fibromyalgia	*N* = 10

Effects of yoga for women with depression (Kinser and Cook)	Examine relationships among depression, stress, inflammation, cellular aging, DNA methylation patterns, and yoga	Women with treatment-resistant depression	*N* = 10

**Table 2:** Results of 4 research studies using the enhanced informed consent process.

Research title	Population	Age range (years)	Ethnicity/race (self-identified)	Enrollment rate (recruitment yield)
Epigenetics and psychoneurologic (PN) symptoms in women with breast cancer (Lyon et al.)	Women diagnosed with early-stage breast cancer	23–71	African American, 50% Caucasian, 50%	80%

Epigenetics and maternal stress (supplement to ongoing intervention trial) (Jallo and Cook)	Healthy pregnant women	18–32	African American, 100%	85%

Stress, immunity and symptoms in fibromyalgia (Menzies and Cook)	Women with fibromyalgia	44–62	Caucasian, 50% African American, 40% Hispanic, 10%	100%

Effects of yoga for women with depression (Kinser and Cook)	Women with treatment-resistant depression	21–61	Caucasian, 44.4% African American, 44.4% Asian, 11.1%	90%

## Appendices

### A. Example of Basic Recruitment Script

Our bodies are made up of cells, much like a beach is made up of small grains of sand. Within every cell we have genes. These genes contain the biologic instructions that tell our cells how to work. A portion of these genes can be turned on or off in response to our exposures or stress experiences and we are trying to learn how this process works. One question that we are asking in this part of the study is if there are some genes that turn on or off in a ______ (fill in the blank with appropriate study aim: for example, mother's body during pregnancy; women with fibromyalgia). We are also interested in trying to learn what environmental or situational events trigger these changes.

One way to picture this concept is to think of our body as a house. In your house there is wiring that is present, much like the way that there our genes present in our body. Some of the items that are plugged into the wiring are always on (like the refrigerator). These items are comparable to the group of genes that we have in our bodies that are also always turned on. However, there are some items that you turn on and off in response to your needs. For example, you may walk into different rooms and flip switches to turn the lights on or off. A portion of these devises are easily changed in response to needs (for example flipping lights on or off). However there are some devises that, once an initial change is made, require more effort to reverse. For example, when a lot of devices are used on one circuit or when there is some sort of power surge you can flip a breaker in a house. To get the electric to work again for that subset of devices you have to identify where the change occurred (which breaker flipped) and then actively flip the switch back to regain the original function. The options for having devices working in your home is a combination of the wiring that is present (our genes), adding devices and turning them on or off (our environment) and then using (or not using) the device, such as using the light to read by, watching the TV, or having a dark room for sleeping (changes in the cells). We are interested in learning about the types of changes that arise in a mother's body during pregnancy (turning devices on and off) and the interactions that influence if these changes occur and if so, for how long.

### B. Frequently Asked Questions

Can you tell if my baby has a problem?

No, we are not looking for or testing for any genetic problems with the baby. Your provider may talk to you about some potential prenatal screening tests for this reason but we are not testing the baby for any genetic conditions.

Could you find anything “bad”?

We are not looking at inherited changes that cause people to have genetic diseases. Instead we are looking at factors that might turn genes on and off in our body's cells. To use our house analogy, we are not looking at how the wiring in the house is designed but rather changes in how the devices plugged into the wiring are used.

Will you provide me with the results of these tests?

No since this is a very new study, we do not know how important any of the findings will be. Because we are just beginning to look at how the body's cells might turn on and off with stress, it is too early to provide you with any individual report or information. By participating in this study you would be providing the first gains in our knowledge about these types of changes.

Will anyone see these results?

The results of this study will not be provided to you or your doctor. They will not be in your health record nor will the results have any effect on your treatment. All of the information gained will be kept strictly confidential to ensure that your privacy is protected.

Will there be any extra costs for these studies?

No, since this is a research project you will not have any costs extra.

Will giving extra blood be harmful to my baby or me?

While the filled tubes look like they have a lot of blood, you are actually providing only about 2 tablespoons of blood. This will not have any bad impact on your health or the health of your baby. For comparison, when people donate blood a pint is collected and even taking that much blood has no bad effects.

If you do not know if there are any changes, why should I participate?

You are correct; at this time we do not know if there will be any changes. That is why this is considered to be a research test. The only way we can gain knowledge is by studying people who are pregnant to learn if these changes occur. Our hope is that if we can identify consistent changes that occur in women with specific types of health concerns during their pregnancy then we can develop methods in the future that might help us to treat women or develop lifestyle changes to help women avoid these problems during their pregnancy.

How will I know about my DNA?

None of this blood work is used to diagnose or treat you. We are looking at relationships of “markers” and symptoms. We do not share your results with anyone. Again, we look at averages and compare those. We will not be giving lab results to you because yours alone is not the primary focus of this work.
